# Supplementary material for: Evidence of a coupled electron-phonon liquid in NbGe2
Source: Nat Commun. 2021 Sep 6;12:5292. doi: 10.1038/s41467-021-25547-x (PMC8421384; doi:10.1038/s41467-021-25547-x)
Supplement: Supplementary file 1 — Supplementary information [file 41467_2021_25547_MOESM1_ESM.pdf]

Supplementary Information: Evidence of a coupled electron-phonon liquid in $NbGe_2$

Hung-Yu Yang[1]*, Xiaohan Yao[1], Vincent Plisson[1], Shirin Mozaffari[2], Jan P. Scheifers[3], Aikaterini Flessa Savvidou[4], Eun Sang Choi[2], Gregory T. McCandless[3], Mathieu F. Padlewski[5], Carsten Putzke[5], Philip J. W. Moll[5], Julia Y. Chan[3], Luis Balicas[2,4], Kenneth S. Burch[1], Fazel Tafti[1]*

[1]*Department of Physics, Boston College, Chestnut Hill, MA 02467, USA*

[2]*National High Magnetic Field Laboratory, Florida State University, Tallahassee, Florida 32310,USA*

[3]*Department of Chemistry and Biochemistry, University of Texas at Dallas, Richardson, TX 75080, USA*

[4]*Department of Physics, Florida State University, Tallahassee, Florida 32306,USA*

[5]*École Polytechnique Fédéral de Lausanne (EPFL), 1015 Lausanne, Switzerland, Laboratory for Atomic and Solid State Physics*

Supplementary Note 1: de Haas-van Alphen Experiments

We used a piezoresistive cantilever technique to study dHvA oscillations in $NbGe_2$ (Supplementary Fig. 1). In this technique, the electrical resistance of the lever is proportional to the Pauli paramagnetic response due to an external magnetic field. The oscillatory part of the data, after removing a smooth background, is plotted as a function of inverse field (H^{-1}) in Supplementary Fig. 1a. The dHvA oscillations are plotted at 9 representative temperatures (between 0.47 and 10.04 K). We preformed a fast-Fourier transform (FFT) with a Hamming window [1] on the dHvA oscillations to obtain the FFT spectra in Supplementary Fig. 1b,c. $NbGe_2$ has both large orbits with frequencies more than 2 kT and small orbits with frequencies less than 1 kT. The thermal damping of FFT peaks fit to a Lifshitz-Kosevich (LK) formula [2]

$$A = A_0 \frac{X}{\sinh X}, \quad X = \frac{\alpha T}{H} m^*, \tag{1}$$

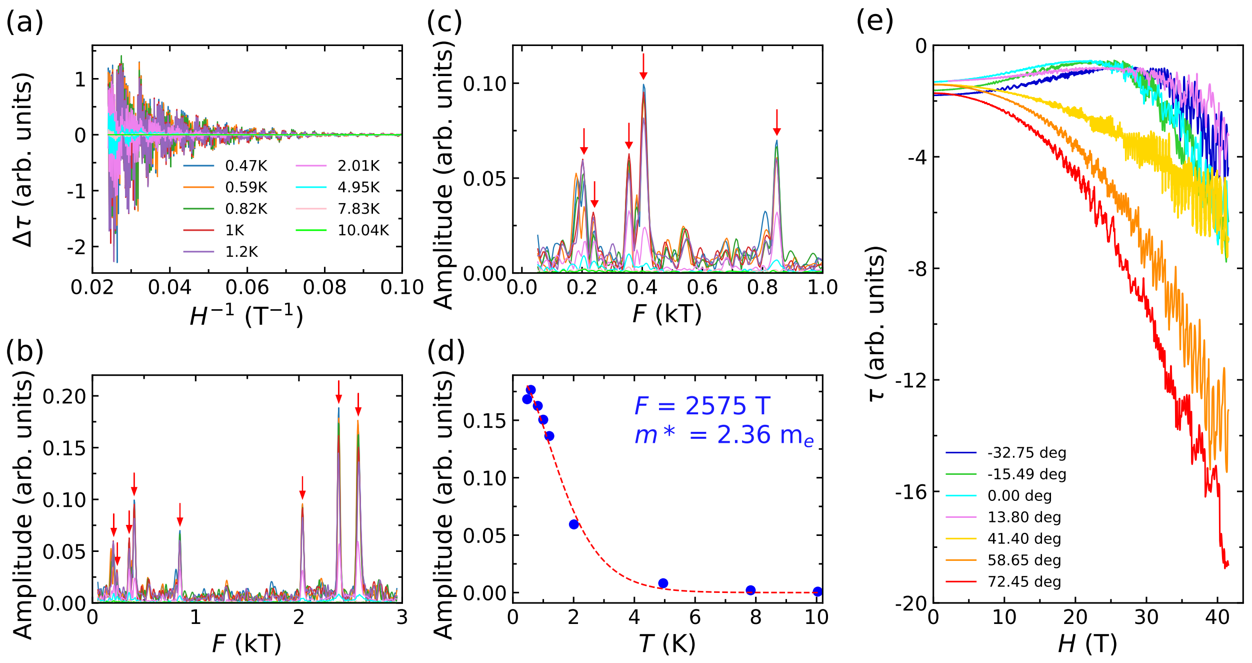

Supplementary Figure 1: **de Haas-van Alphen oscillations.** (a) The oscillatory part of torque data as a function of inverse field. (b) FFT spectra at different temperatures. The characteristic frequencies of $NbGe_2$ are marked by red arrows. (c) A magnified view of the FFT spectra at low frequencies. (d) An example of the Lifshitz-Kosevich analysis for $F = 2386$ T. (e) Angle dependence of dHvA data.

Supplementary Table 1: **Fermi surface parameters.** The experimental dHvA frequencies and effective masses are listed as $F_{\rm exp}$ and $m^*_{\rm exp}$, while the theoretical values are denoted as $F_{\rm DFT}$ and $m^*_{\rm DFT}$. Notice that the experimental masses are approximately three times larger than the theoretical ones (evidence of ph-el interactions). The mild mismatch between experimental and theoretical frequencies is due to an uncertainty in the angle between the sample and the magnetic field (10-20°).

$F_{\rm exp}$ (T)	206.5	241.7	355.9	404.2	848.0	2034	2386	2575
$m^*_{\rm exp}/m_e$	1.71	1.17	1.55	2.45	1.87	2.28	2.45	2.36

$F_{\rm DFT}$ (T)	86.5	115.9	135.4	290.4	445.4	544.6	1981	2088	2103	2230	2593
$m^*_{\rm DFT}/m_e$	0.23	0.23	0.39	0.55	0.53	0.61	0.75	0.78	0.85	0.86	1.17

where $\alpha = 2\pi^2 k_B m_e/e\hbar$ is a constant comprising the Boltzmann constant k_B, bare electron mass m_e, and reduced Plank constant $\hbar$. H is the magnetic field window of FFT, m^* is the effective mass in units of m_e, and A_0 is a fit parameter corresponding to the peak amplitude at zero temperature. By fitting the temperature dependence of each FFT peak to Supplementary Eq. 1, we extracted the effective mass of the quasiparticle on each cyclotron orbit. An example of such a fit is shown in Supplementary Fig. 1d, where the blue circles represent the experimental data (FFT peak amplitude at different temperatures) and the red dashed line represents the LK fit. The experimental values of m^* in the main Supplementary Fig. 1d were all obtained from such LK fits. All of the observed frequencies and their effective masses in $NbGe_2$ are listed in Supplementary Table 1. For the above analyses, we used dHvA data with the field at 41.4° with respect to the hexagonal planes of the sample, because most of the ferequencies were visible at that angle. Figure 1e shows the results of a screening test, where we obtained dHvA data at several angles and decided to use the angle with the richest pattern of quantum oscilaltions (41.4°).

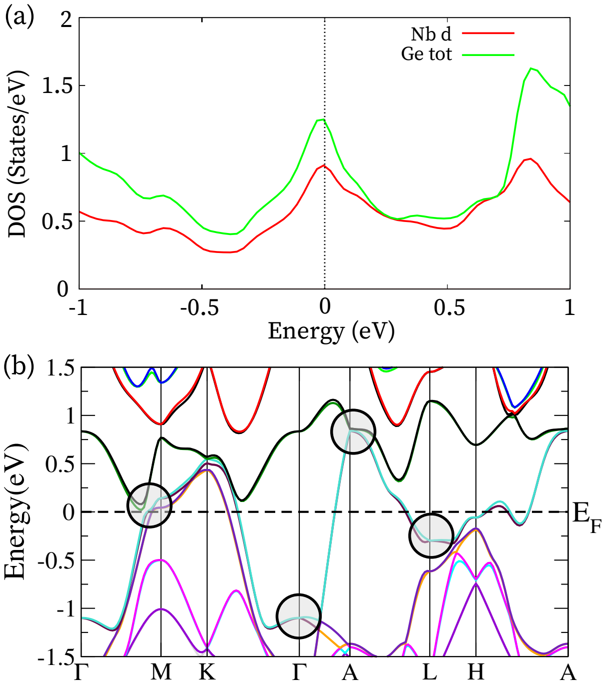

Supplementary Figure 2: **Density of states and band structure.** (a) Partial density of states, showing a mix of Nb-d and Ge-p and s orbitals at the Fermi level. (b) Band structure of $NbGe_2$ with the Kramers-Weyl points at M, Γ, A, and L points.

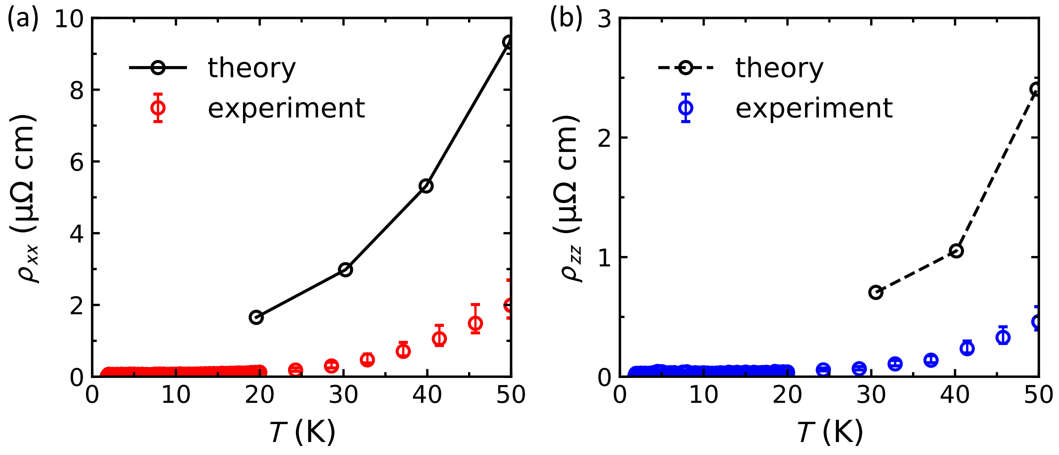

Supplementary Figure 3: **Comparing the experimental and theoretical resistivity curves.** Comparison between the theoretical calculation and experimental data (from Ref. [5]) for (a) ρ_{xx} and (b) ρ_{zz} as a function of temperature below 50 K.

Supplementary Note 2: Band Structure

Figure 2a shows the partial density of states (DOS) at the Fermi level with an equal contribution from Nb-d and Ge-p and s orbitals. We show the band structure of both $NbGe_2$ in Supplementary Fig. 2b calculated in a PBE+SOC scheme using the WIEN2k code [3]. Because the material has a chiral crystal structures, it supports Kramers-Weyl points by symmetry [4,5]. Such points are highlighted by circles at the Γ, M, A, and L points in Supplementary Fig. 2b. However, the transport properties of these compounds are dominated by large and dispersive bands that cross E_F and create a Fermi surface with large orbits (Supplementary Fig. 1).

Supplementary Note 3: Resistivity Anisotropy

Figure 3 shows that the discrepency between the theoretical calculations of Ref. [5] and the experimental data is not limited to high temperatures. It continues to low temperatures, well below the cross-over temperature for the phonon-drag process ($\Theta = 155$ K).

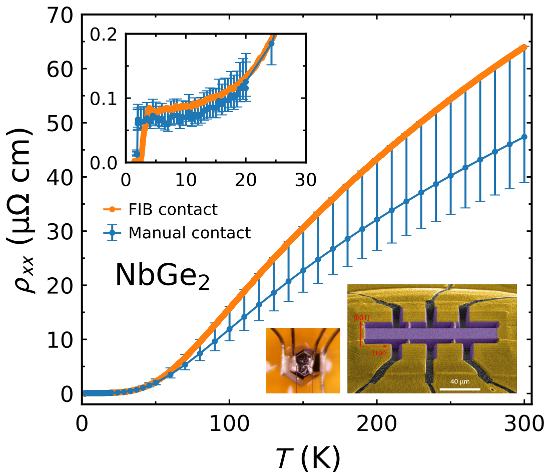

Supplementary Figure 4: **Data from a mesoscopic device.** The resistivity of a mesoscopic device (bottom right inset) made from $NbGe_2$ is compared to the resistivity of a bulk crystal (bottom center inset).

The resistivity data in the main Fig. 2 text and Supplementary Fig. 3 were obtained from single crystals with hand-made contacts. Thus, there is an uncertainty in the geometric factor that converts electrical resistance to resistivity (width$\times$height/length). To ensure this uncertainty is not the source of discrepancy between theory and experiment, we have also measured resistivity on mesoscopic devices made by FIB technology (Supplementary Fig. 4) with geometric errors less than 5% (i.e. $\Delta L/L$, $\Delta W/W$, $\Delta t/t$ <0.05). The comparison between the data from conventional contacts and mesoscopic device in Supplementary Fig. 4 shows an agreement within the error bars and confirms the discrepancy between the theoretical results of Ref. [5] and our experiments.

Supplementary Note 4: Phonon drag and heat capacity

In the main text (Fig. 2c), we showed that a phonon-drag model gives the best fit to the resistivity data. This is confirmed in Supplementary Fig. 5a which is a semilog (Arrhenius) plot of the resistivity versus temperature. We also explained in the main text that the coefficients of a power-law fit, $\rho_{xx} = \rho_0 + AT^2 + BT^5$, do not make physical sense when combined with the Sommerfeld

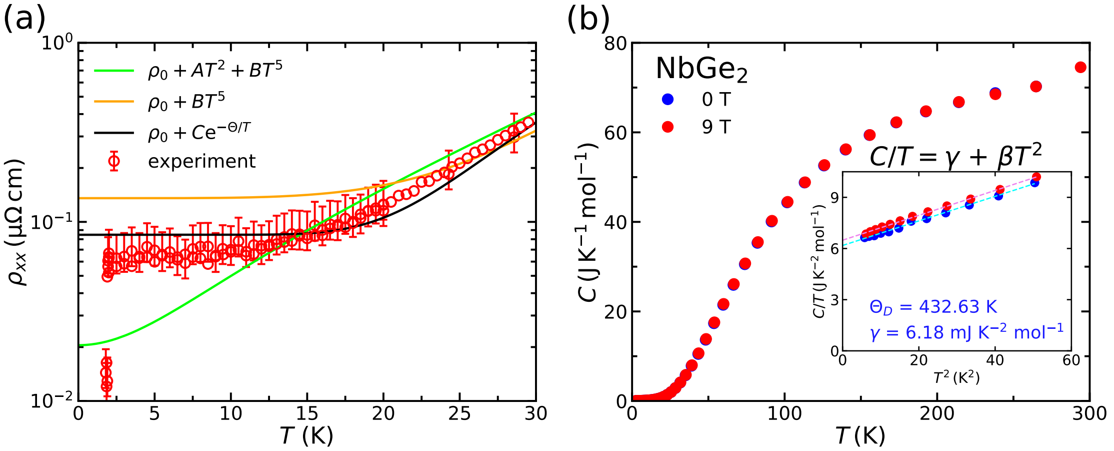

Supplementary Figure 5: **Phonon drag and Debye model.** (a) Comparing three model fits to $\rho(T)$ on a semilog (Arrhenius) plot. The data is best described by a phonon-drag (exponential) model. (b) Measurements of the heat capacity are performed at both $H = 0$ and 9 T. A fit to the Sommerfeld-Debye model is presented in the insets.

coefficient γ from the heat capacity data to compute the Kadowaki-Woods ratio $R_{\mathrm{KW}} = \frac{A}{\gamma^2}$. Figure 5b shows the results of our heat capacity measurements. By fitting the low-temperature data to a Sommerfeld-Debye model $C/T = \gamma + \beta T^2$ (inset of Supplementary Fig. 5b), we evaluate the Sommerfeld coefficient $\gamma = 6.18$ mJ mol^{-1}K^{-2} and Debye temperature $\Theta_D = 433$ K (using $\beta = \frac{12\pi^4}{5}\frac{nk_B}{\Theta_D^3}$). A small γ is consistent with $NbGe_2$ being a non-magnetic system with weak electronic correlations. Using $k_B\Theta_D = \hbar c k_D$, we also evaluate the sound velocity $c = 5292$ m/s.

Supplementary Note 5: X-ray Diffraction

The crystallographic refinement parameters are summarized in Supplementary Table 2. The refinement was performed in the space group #180 with Nb and Ge atoms in the $3d$ and $6j$ Wyckoff sites, respectively, with coordinates ($\frac{1}{2}$, 0, $\frac{1}{2}$) and (0.1638(1), 0.3275(2), $\frac{1}{2}$). The isotropic displacement parameters were 0.005(2) and 0.006(1) Å^2 for Nb and Ge, respectively.

Supplementary Table 2: **Single crystal X-ray diffraction refinement parameters.** Crystal structure refinement of $NbGe_2$ as obtained from single crystal X-ray diffraction.

Formula	$NbGe_2$
Formula weight (g/mol)	238.09
Space group	$P6_222$ (#180)
Lattice parameters (Å)	$a = b = 4.9677(6)$
	$c = 6.7853(19)$
Unit cell volume (Å^3)	145.01(5)
Z	3
Density (g/cm^3)	8.179
Θ range	4.738 – 33.310
Number of reflections	6430
Unique reflections	192
Flack parameter (classical, Parsons)	-0.03(4), 0.01(4)
GOF	0.938
$R_1[F^2 > 2\sigma(F^2)]$	0.0159
$wR_2(F^2)$	0.0973
$\Delta\rho_{max}$ (e/Å^3)	0.768
$\Delta\rho_{max}$ (e/Å^3)	-0.579

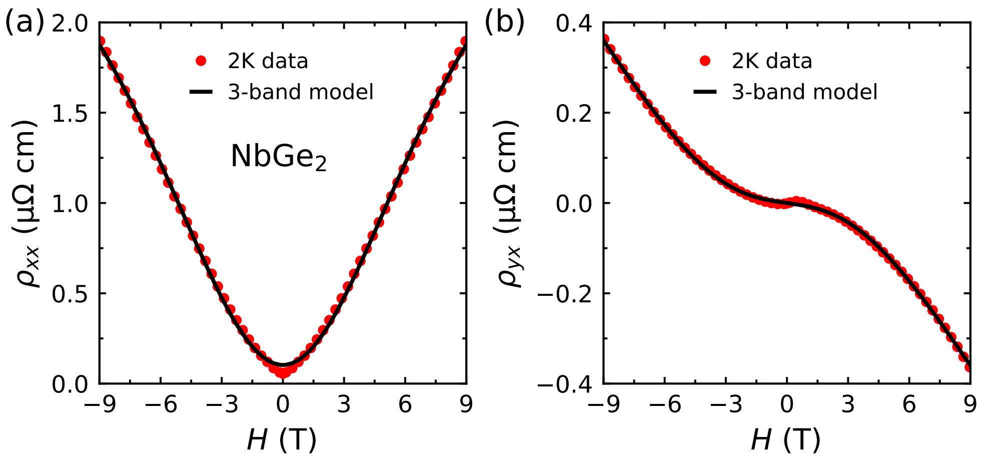

Supplementary Figure 6: **Multiband fits.** A three-band model is fitted simultaneously to both resistivity (ρ_{xx}) and Hall effect (ρ_{yx}) in $NbGe_2$.

Supplementary Note 6: Carrier Concentration and Mobility

By performing a multiband fit to the longitudinal and transverse resistivity data (ρ_{xx} and ρ_{xy}), we estimate the electron and hole concentrations in excess of 10^{21} cm^{-3} in $NbGe_2$ (Supplementary Table 3). Thus, $NbGe_2$ is classified as a metal, not a semimetal as mentioned in previous works [5]. To extract phenomenological carrier concentrations and mobilities of $NbGe_2$, we fit a multiband model with the following expressions to the transport data [6]:

$$\rho_{xx} = \frac{\sum_i \frac{\sigma_i}{1+\mu_i^2 B^2}}{\left(\sum_i \frac{\sigma_i}{1+\mu_i^2 B^2}\right)^2 + \left(\sum_i \frac{\sigma_i \mu_i}{1+\mu_i^2 B^2}\right)^2 B^2}, \tag{2}$$

$$\rho_{yx} = \frac{\left(\sum_i \frac{\sigma_i \mu_i}{1+\mu_i^2 B^2}\right) B}{\left(\sum_i \frac{\sigma_i}{1+\mu_i^2 B^2}\right)^2 + \left(\sum_i \frac{\sigma_i \mu_i}{1+\mu_i^2 B^2}\right)^2 B^2}, \tag{3}$$

where $\sigma_i = n_i e \mu_i$ is the conductivity of the band i, n_i is carrier concentration, μ_i is mobility, and the summation i runs over all the bands considered. We are assuming a minimal model with two electron bands and one hole band, and fit both ρ_{xx} and ρ_{xy} data to the above expressions simultaneously. Figure 6 shows a decent agreement between the model and experimental data, and the high carrier concentrations and mobilities (Supplementary Table 3.) are consistent with the

Supplementary Table 3: **Multiband fit parameters.** The carrier concentrations and mobilities, which are fitting coefficients of the three-band model (Supplementary Fig. 6), are reported here.

Band character	n (cm^{-3})	μ (cm^{2}V^{-1}s^{-1})
electron	4.45×10^{21}	7020
hole	4.14×10^{21}	6650
electron	1.00×10^{22}	174

large Fermi surface and low residual resistivity in $NbGe_2$.

Supplementary Note 7: Sample quality

$NbGe_2$ crystals were grown using a chemical vapor transport (CVT) method. We have improved the sample quality iteratively by changing the amount of transport agent (iodine), the tube length, the heating sequence, and the magnitude and direction of temperature gradient. The resistivity data from 9 samples are presented in (Supplementary Fig. 7) with increasingly larger residual resistivity ratio (RRR) from S1 to S9. Although several parameters can be tuned during a CVT growth, we found that the most efficient way of improving sample quality was by reducing the temperature gradient to be less than 10 °C while the furnace hot zone was at 900 °C or higher. For the best $NbGe_2$ sample (S9) used in this study, the details of the growth is as follows: Nb and Ge powders were mixed and ground with the ratio Nb:Ge=1:2. 500 mg of the powder mixture was transferred to a 3.5-inch long, small-sized silica tube. 10 mg of I_2 was added, and then the tube was sealed under vacuum. The tube was placed in a box furnace, heated up to 900 °C at 3 °C/min, and dwelled at that temperature for 1 month.

We performed energy dispersive X-ray spectroscopy (EDX) on most of the samples in Supplementary Fig. 7 and summarized the results in Supplementary Table 4 for 4 batches of samples. We have measured the chemical composition of a few samples in each batch and found similar Nb to Ge ratios despite different growth conditions. Therefore, the variations of RRR in Supplemen-

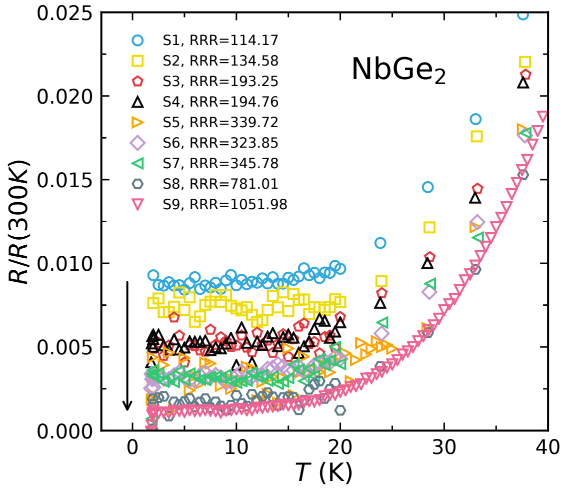

Supplementary Figure 7: **Sample quality.** Normalized resistance $R/R(300\mathrm{K})$ is plotted as a function of temperature for 9 samples grown under slightly different conditions. The residual resistance is systematically reduced by improving sample quality. This is reflected in the increasing residual resistivity ratio (RRR) from S1 to S9.

tary Fig. 7 are due to varying degrees of extended defects (such as dislocations) instead of local defects (e.g. vacanies and inter-site disorder).

Supplementary Table 4: **Energy disperisive x-ray analysis.** Average of the atomic percentage of Nb and Ge in 4 different sample batches. The labels correspond to those in Supplementary Fig. 7.

Batch	RRR	Nb (%)	Ge (%)
S1	114	34.33(28)	65.64(33)
S4	195	34.23(55)	65.77(55)
S7	346	34.26(60)	65.74(60)
S8	781	34.32(33)	65.68(33)

References

1. Harris, F. J. On the use of windows for harmonic analysis with the discrete Fourier transform. *Proceedings of the IEEE* **66**, 51–83 (1978).

2. Shoenberg, D. *Magnetic Oscillations in Metals* (Cambridge University Press, Cambridge, 2009), 1st edition edn.

3. Blaha, P. *et al.* *WIEN2K an augmented Plane wave + Local Orbitals program for calculating crystal properties* (Karlheinz Schwarz, Techn. Universität, Wien, Austria, 2018).

4. Chang, G. *et al.* Topological quantum properties of chiral crystals. *Nature Materials* **17**, 978–985 (2018). URL `https://www.nature.com/articles/s41563-018-0169-3`.

5. Garcia, C. A. C., Nenno, D. M., Varnavides, G. & Narang, P. Anisotropic phonon-mediated electronic transport in chiral Weyl semimetals. *arXiv:2012.09207 [cond-mat]* (2020). URL `http://arxiv.org/abs/2012.09207`.

6. Ziman, J. M. *Principles of the Theory of Solids* (Cambridge University Press, Cambridge, 1972), 2 edn. URL `https://www.cambridge.org/core/books/principles-of-the-theory-of-solids/F9E87699164B7094168277D4867EE4FC`.
